# Long-term spatiotemporal stability and dynamic changes in the haemoparasite community of spiny mice (*Acomys dimidiatus*) in four montane wadis in the St. Katherine Protectorate, Sinai, Egypt

**DOI:** 10.1186/s13071-016-1471-z

**Published:** 2016-04-08

**Authors:** Mohammed Alsarraf, Małgorzata Bednarska, Eman M. E. Mohallal, Ewa J. Mierzejewska, Jolanta Behnke-Borowczyk, Samy Zalat, Francis Gilbert, Renata Welc-Falęciak, Agnieszka Kloch, Jerzy M. Behnke, Anna Bajer

**Affiliations:** Department of Parasitology, Institute of Zoology, Faculty of Biology, University of Warsaw, 1 Miecznikowa Street, 02-096, Warsaw, Poland; Desert Research Center, Cairo, Egypt; Department of Forest Phytopathology, Faculty of Forestry, Poznań University of Life Sciences, Poznań, Poland; Department of Zoology, Suez Canal University, Ismailia, Egypt; Faculty of Medicine & Health Sciences, School of Biology, University of Nottingham, Nottingham, UK; Department of Ecology, Institute of Zoology, Faculty of Biology, University of Warsaw, 1 Miecznikowa Street, 02-096, Warsaw, Poland

**Keywords:** *Acomys dimidiatus*, *Acomys russatus, *haemoparasites, *Haemobartonella*, *Bartonella*, *Hepatozoon*, *Trypanosoma*, *Babesia*, Species-richness, Prevalence, Abundance, Sinai, Drought, Between year variation

## Abstract

**Background:**

Long-term field studies of parasite communities are rare but provide a powerful insight into the ecological processes shaping host-parasite interactions. The aim of our study was to monitor long-term trends in the haemoparasite communities of spiny mice (*Acomys dimidiatus*) and to identify the principal factors responsible for changes over a 12 year period.

**Methods:**

To this end we sampled four semi-isolated populations of mice (*n* = 835) in 2000, 2004, 2008 and 2012 in four dry montane valleys (wadis) located in the Sinai Massif, Egypt.

**Results:**

Overall 76.2 % of spiny mice carried at least one of the five haemoparasite genera (*Babesia, Bartonella, Haemobartonella, Hepatozoon, Trypanosoma*) recorded in the study. Prevalence of haemoparasites varied significantly between the sites with the highest overall prevalence in Wadi Tlah and the lowest in W. El Arbaein, and this changed significantly with time. In the first two surveys there was little change in prevalence, but by 2008, when the first signs of a deepening drought in the region had become apparent, prevalence began to drift downwards, and by 2012 prevalence had fallen to the lowest values recorded from all four sites over the entire 12-year period. The overall mean species richness was 1.2 ± 0.03, which peaked in 2004 and then dropped by more than 50 % by 2012. Species richness was highest among mice from Wadi Tlah and peaked in age class 2 mice (young adults). Site was the most significant factor affecting the prevalence of individual parasite species, with *Trypanosoma acomys* and *Hepatozoon* sp. occurring mainly in two wadis (W. Tlah & W. Gharaba). In four of the five genera recorded in the study we observed a significant drop in prevalence or/and abundance since 2004, the exception being *Hepatozoon* sp*.*

**Conclusions:**

During the 12-year-long period of study in the Sinai, we observed dynamic changes and possibly even cycles of prevalence and abundance of infections which differed depending on parasite species. Although the exact reasons cannot be identified at this time, we hypothesize that the effects of a 15-year-long scarcity of rainfall in the local environment and a fall in host densities over the period of study may have been responsible for a drop in transmission rates, possibly by a negative impact on vector survival.

**Electronic supplementary material:**

The online version of this article (doi:10.1186/s13071-016-1471-z) contains supplementary material, which is available to authorized users.

## Background

Long-term field studies of parasite communities provide a powerful insight into ecological and evolutionary processes shaping host-parasite interactions over time. Small mammals, especially rodents, are good model hosts for such studies because their populations are abundant, heterogenous and highly dependent on environmental factors, including food availability and climatic conditions [1–3]. The high heterogeneity and the dynamic between- and within-year variation of rodent populations allow also investigation of the relative contribution of a range of quantifiable intrinsic and extrinsic factors underlying some of the dominant patterns of variation in parasitic infections observed in the field [4–7]. Each rodent community can be regarded as comprising a set of different functional subgroups including, for example, settled, territorial adults of both sexes and mobile juveniles, which may differ in their exposure and susceptibility to infection [8].

Climatic conditions are believed to play a crucial role in shaping plant or animal communities in different habitats, including those living in arid environments such as the deserts of the Middle East, and are likely also to have a major impact on the parasite communities of the indigenous hosts. However, other than in short-term catastrophic events (e.g. earthquakes, intense storms etc.), the influence of climatic changes is likely to be slow over a prolonged period of time and hence long-term ecological studies are essential to identify links between changing climatic conditions and disease.

In earlier studies we have shown that the haemoparasite communities of *Acomys dimidiatus* vary markedly between subpopulations of spiny mice living in four distinct dry semi-isolated desert valleys (wadis) in the Sinai mountains of Egypt. These four wadies are segregated from each other by natural barriers [9, 10] and they differ in altitude but ecologically they show many similarities although distinct differences have also been recorded [11]. Such isolated or semi-isolated subpopulations of animals may differ in the stresses to which they are subjected in each site, including pathogens, and hence may experience different selection pressures created by the specific conditions in their home range (the geographical mosaic theory of co-evolution; [12]). Haemoparasites in particular are likely to be an important source of selective pressure on hosts because they are often associated with pathogenicity (e.g. acute babesiosis, trypanosomiasis; [13–15]) and hence, resistance/tolerance of such infections confers enormous fitness benefits [16–18]. In this context it is pertinent that in our first survey in 2000, the haemoparasite community of the spiny mice living in these wadies were more diverse [10] than in our concurrent and subsequent studies on the haemoparasites of common or bank voles in Poland [4, 19–21] or in other studies on rodents [22–24].

We continued to monitor the haemoparasites of the same spiny mouse populations in 2004, 2008 and 2012 in order to assess the stability of the epidemiological patterns that were observed in 2000. Here, building on the resources collected in these four expeditions to the Sinai and the resultant database on natural infections in wild spiny mice, we report on the spacio-temporal stability of some haemoparasite species carried by *A. dimidiatus* and on the dynamic changes in others in our study sites. Detailed morphometric data on each animal also allowed the effect of host intrinsic factors on haemoparasites to be assessed. We predicted that the effects of host age and sex would be consistent and repeatedly observable in successive surveys, showing little between-year variation in magnitude of the effect due to co-evolution of the hosts and parasites involved. Haemoparasites are vector-borne pathogens (VBP) and in short-lived host species, such as spiny mice, we would expect a significant increase in the prevalence of VBP with host age, as the probability of being infested with ticks or fleas carrying VBP increases also with host age. However, host immune responses to each of the haemoparasites differ, and where host-protective immunity is generated, we would also expect both prevalence and abundance of infection to decline in the oldest age class [25]. We predicted that extrinsic factors (unique abiotic conditions associated with certain wadi and/or particular years of study) would have a greater influence resulting in repeatable patterns (for between-site differences) or distinct between-survey dynamics (for between-year differences). While abiotic conditions are largely ‘unpredictable’ for both hosts and parasites, the climatic changes in the Sinai have been well documented [26–28] and in this long-term study of the parasites of spiny mice we had an opportunity to observe the impact of decreasing water availability on host and parasite populations/communities. Egypt is the most arid country on Earth [29], classified as ‘hyper-arid’ in climatology. Because of the alarming reduction in the water supply in the montane wadies of S. Sinai as a consequence of a long period (15 years) of no or only very low rainfall and the resulting increasing aridity of the local environment with associated loss of arable land (Bedouin gardens), clearly apparent during the expeditions in 2008 and 2012 (compared with the first two expeditions in 2000 and 2004), we expected marked differences in parasite community structure over this period of 12 years. For parasite isolates obtained in 2004–2012 we carried out also preliminary molecular characterization and phylogenetic analyses. Our data provide a novel insight into the ecology of the haemoparasites of rodent hosts living in semi-isolated, hyper-arid habitats, about which little is currently known.

## Methods

### Field studies in Sinai, Egypt

Fieldwork was conducted over 4–5 -week periods in August-September in 2000, 2004, 2008 and 2012 and was based at the Environmental Research Centre of Suez Canal University (2000, 2004) or at Fox Camp (2008, 2012) in the town of St Katherine, South Sinai, Egypt. Trapping was carried out in four montane wadis (dry valleys) in the vicinity of St Katherine. The local environment and general features of the four study sites (Wadi ElArbaein, W. Gebal, W. Tlah, W. Gharaba), as well as their spatial relationships with one another, have been described elsewhere [9]. At each site, rodents were caught live in Sherman traps, placed selectively among the rocks and boulders around walled gardens and occasionally along the lower slopes of wadis. These were set out at dusk, and inspected in the early morning before exposure to direct sunlight. All traps were brought into the local or main camp, where the animals were removed, identified and processed. Traps were re-set the following evening.

The three most abundant rodent species (*A. dimidiatus, A. russatus, D. dasyurus*) were sampled- sexed, weighed, measured and scrutinized for obvious lesions as described by Behnke et al. [9]. Ectoparasites visible during field examination were removed and placed in 70 % ethanol. Blood and faecal samples were taken and animals were then either fur marked individually or ear clipped and released close to the point of capture, or returned to the main camp at St Katherine for autopsy.

Animals were allocated to three age classes, principally on the basis of body weight and nose-to-anus length. For male and non-pregnant female mice separately, these two measurements were reduced by principal components analysis, and principal component 1 (for males Eigen value = 1.87 and accounted for 93.5 % of variance and for non-pregnant females the Eigen value was 1.83, accounting for 91.4 % of variance) together with observations recorded for each animal in the field (for males whether scrotal or non-scrotal, for females whether lactating, perforate or pregnant), was used to guide allocation of animals to three age classes. Full details of the methods used and statistical verification of this approach are given in Behnke et al. [9] (all means are cited ± one standard error). Age class 1 comprised the youngest animals, mostly weanlings and very young non-reproductively active juveniles (mean weight for males = 17.8 g ± 0.40, *n* = 74; females = 19.5 g ± 0.31, *n* = 116), age class 2 comprised juveniles and young adults (mean weight for males = 28.1 g ± 0.29, *n* = 114, non pregnant females = 27.4 ± 0.30, *n* = 104; pregnant females = 28.8 ± 2.49, *n* = 3), and age class 3 comprised the adult and oldest animals in the study (mean weight for males = 41.5 ± 0.42, *n* = 184; non-pregnant females = 40.6 g ± 0.44, *n* = 182; pregnant females = 47.4 ± 1.09, *n* = 58).

### Blood collection and DNA extraction

Thin blood smears were prepared from drops of blood taken from the retro-orbital plexus using heparinized capillary tubes of animals lightly anaesthetized with ether during examination in the field and from the heart of those that were autopsied. Blood smears were air-dried, fixed in absolute methanol and stained for 1 h in Giemsa stain in buffer at pH 7.2. Each smear was examined under oil immersion (×1000 magnification). Parasites were counted in 200 fields of vision. Microscopical observation of stained blood smears was used as the only detection method for study in 2000 and for *Babesia* spp. In subsequent expeditions, in addition to blood smears, molecular techniques were used for species identification of *Bartonella*, *Hepatozoon* and *Trypanosoma* but confined to samples that were positive by microscopical observation in 2004 and 2008, and as the diagnostic method for all samples in 2012. Blood from the tail vein was collected on FTA classic cards (Whatman, UK) for the long-time preservation of DNA. From the culled animals, 200 μl of whole blood were also collected into 0.001 M EDTA and frozen at -20 °C.

Genomic DNA was extracted from whole blood using DNAeasy Blood & Tissue kit (Qiagen, USA) or AxyPrep MiniPrep Blood kit (AxyGen, USA) and stored at a temperature of -20 °C. DNA from FTA cards was cleaned with FTA purification Reagent (Whatman, UK) according to the manufacturer’s instructions.

### Molecular characterization

The extracted DNA was subjected to specific PCRs as described in detail in Bajer et al. [4]. The primers and cycling conditions used in this study are listed in a table (Additional file 1). Reactions were performed in 1× PCR buffer, 0.2 U *Taq* polymerase, 1 μM of each primer and 2 μl of the extracted DNA sample. Negative controls were conducted in the absence of template DNA. PCR products were subjected to electrophoresis on a 1.5 % agarose gel, stained with Midori Green stain (Nippon Genetics GmbH) and sequenced by a private company (Genomed S.A., Poland).

### Genotyping and phylogenetic analysis

#### *Bartonella* sp.

One *Bartonella* isolate obtained from *A. dimidiatus* from W. Tlah in 2004 was genotyped by the amplification and sequencing of a 333-bp fragment of the *rpoB* region [30].

#### *Hepatozoon*

Thirty nine isolates derived from *A. dimidiatus* and *A. russatus* in 2004, 2008 and 2012 from all sites (Table 1) were investigated by the analysis of a 660 bp 18S rRNA gene fragment [31]. First, all obtained sequences were aligned using MEGA v. 6.0. The phylogenetic analyses including our sequences (660 bp) and sequences of *Hepatozoon* spp. deposited in the GenBank database were conducted in MEGA v. 6.0 [32]. A representative tree for 18S rDNA sequences was obtained using the Maximum Likelihood method and a Tamura 3-parameter (I + G) model.

**Table 1:** *Hepatozoon* isolates/variants by the host, site and year of study

		2004				2008				2012				Total
		Arbaein	Gebal	Gharaba	Tlah	Arbaein	Gebal	Gharaba	Tlah	Arbaein	Gebal	Gharaba	Tlah	
*A. dimidiatus*	Variant A	0	1	1	0	4	3	3	4	5	0	2	9	32
*A. russatus*	Variant A	0	0	0	0	1	1	2	0	0	0	0	1	5
	Variant B	0	0	1	0	0	0	0	0	0	0	0	1	2
Total		0	1	2	0	5	4	5	4	5	0	2	11	39

#### *Trypanosoma*

Forty five isolates derived from *A. dimidiatus* from 2004, 2008 and 2012 from W. Gebal (*n* = 1), W. Gharaba (*n* = 15) and W. Tlah (*n* = 29) were investigated by the analysis of a 520 bp 18S rRNA gene fragment [33]. The phylogenetic analyses including our sequences (520 bp) and other sequences of *Trypanosoma* spp. deposited in the GenBank database were conducted in MEGA v. 6.0 [32]. A representative tree for 18S rDNA sequences was obtained using the Maximum Likelihood method and a Tamura 3-parameter (I + G).

### Statistical analysis

Prevalence (percentage of animals infected) was estimated based on microscopical observations and values are reported with the 95 % confidence limits, calculated by bespoke software based on the tables of Rohlf and Sokal [34]. The intensity of infection in each animal was quantified as the number of infected red blood cells (iRBC) [for *Babesia, Bartonella, Haemobartonella* (*Mycoplasma*)] or parasites (for *Trypanosoma, Hepatozoon*) in 200 fields of vision at ×1000 magnification and mean abundance is the average of this measure, including all the sampled animals whether infected or not. Species richness was calculated as the number of different haemoparasite species in each animal. When samples were only positive by PCR (in 2012), an intensity of 1 iRBC/1 parasite in 200 fields of vision was implemented into quantitative statistical analysis.

The statistical approach adopted has been documented comprehensively in our earlier publications [4, 6, 7]. For analysis of prevalence we used maximum likelihood techniques based on log - linear analysis of contingency tables in the software package IBM SPSS (version 21.0.0, IBM Corp). Initially, full factorial models were fitted, incorporating as factors SEX (2 levels, males and females), AGE (3 levels), YEAR of study (4 levels, each of the four surveys), and SITE (4 levels, the four study sites). The presence of parasites was implemented as INFECTION and was considered as a binary factor (2 levels, present or absent). These factors were fitted initially to all models that were evaluated. For each level of analysis in turn, beginning with the most complex model, involving all possible main effects and interactions, those combinations that did not contribute significantly to explaining variation in the data were eliminated in a stepwise fashion beginning with the highest level interaction (backward selection procedure). A minimum sufficient model was then obtained, for which the likelihood ratio of *χ*^2^ was not significant, indicating that the model was sufficient in explaining the data. The importance of each term (i.e. interactions involving INFECTION) in the final model was assessed by the probability that its exclusion would alter the model significantly and these values relating to interactions that included INFECTION are given in the text. The remaining terms in the final model that did not include INFECTION are not given but can be made available from the authors on request.

For analyses of quantitative data we used general linear models (GLM) with normal errors implemented in R version 2.2.1 (R Core Development Team) and the residuals were checked for approximate Gaussian distribution. When the residuals failed to meet the requirements of Gaussian model we explored models based on log_10_ (X + 1) transformed data and generalised linear models with negative binomial or Poisson error structures. Full factorial models that converged satisfactorily were simplified using the STEP procedure and tested for significance using deletion of terms beginning with the highest order interaction by comparing models with or without that interaction. Changes in deviance (DEV) are given for models based on Poisson errors (interpreted as Chi^2^ values), for models based on Gaussian errors we give F and for those based on negative binomial errors the likelihood ratio (LR). Minimum sufficient models were then fitted (all significant interactions and main effects plus any main effects that featured in interactions) and the process was repeated to obtain values for changes in deviance, test statistics and probabilities. Finally, if the data did not meet the assumptions of parametric tests, we employed non-parametric tests (Kruskal- Wallis test and the Mann - Whitney *U*-test).

### Ethical issue

Rodents from St Katherine National Protectorate were sampled by agreement with the St Katherine National Protectorate authorities obtained for each set of fieldwork. A maximum of 40 % of the captured rodents from each site were culled by agreement with the St Katherine National Protectorate authorities.

## Results

### Molecular identification of parasite species (2004, 2008 and 2012)

#### *Bartonella* sp.

One *Bartonella* sp. isolate obtained from *A. dimidiatus* from W. Tlah in 2004 was successfully genotyped by the amplification and sequencing of a 333-bp fragment of the *rpoB* region. Comparison with the GenBank database revealed that the isolate showed the highest sequence homology (99.37 %; 317/319 bp) with *Bartonella acomydis* strain KS2-1 obtained from *A. russatus* (AB529942). This reference strain was identified in the golden spiny mouse imported from Egypt to Japan as an exotic pet [35]*.*

#### *Hepatozoon* sp.

Thirty nine *Hepatozoon* sp. isolates obtained from 2004, 2008 and 2012 from all sites and two host species, *A. dimidiatus* and *A. russatus* (Table 1), were genotyped by sequencing of a 660 bp fragment of the 18S rRNA gene. Two genetic variants of *Hepatozoon* were identified, variant A and B. Variant A was widespread, identified in 32 *A. dimidiatus* and 5 *A. russatus* at all sites (Table 1). Four representative sequences of this variant were deposited in the GenBank database under the accession numbers KT337467, KT337468, KT337471 and KT337472. Variant B was identified only in 2 *A. russatus* (1 from W. Gharaba, 2004 and 1 from W. Tlah, 2012). Both sequences of this variant were deposited in the GenBank database under the accession numbers KT337469 and KT337470.

A BLAST search in the GenBank database revealed, that variant A showed the highest sequence homology (96.23 %) to *Hepatozoon* sp. AO5 from the olive grass mouse, *Abrothrix olivaceus,* from Chile (FJ719818) and 96.06 % sequence homology to *H. ayorgbor* from the royal python, *Python regius,* from Ghana (EF157822). Variant B showed the highest sequence homology (96.75 %) to *H. ayorgbor* from *P. regius* (EF157822) and 97.09 % to *Hepatozoon* sp. AO5 from *A. olivaceus* (FJ719818). Both variants showed a lower sequence homology with *Hepatozoon* isolates from jerboas *Jaculus orientalis* and *J. jaculus* (95.5–96.5 %) [36].

**Fig. 1:**
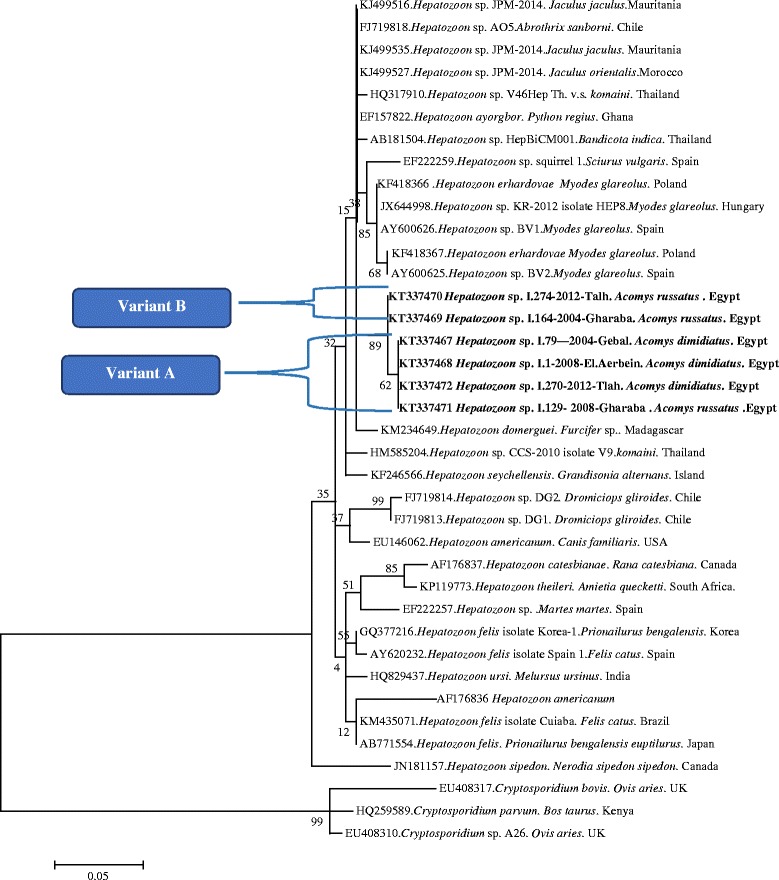
The evolutionary history of *Hepatozoon,* based on a fragment of the 18S rRNA gene, was inferred using the Maximum Likelihood method and a Tamura 3-parameter (I + G). The percentage of replicate trees in which the associated taxa clustered together in the bootstrap test (1000 replicates) are shown next to the branches. The analysis involved 38 nucleotide sequences. All positions containing gaps and missing data were eliminated. The nucleotide sequence of *Cryptosporidium parvum*, *C. ovis* and *C. bovis* were used as outgroups. Evolutionary analyses were conducted in MEGA6.0

Alignment of our two *Hepatozoon* variants revealed a difference of 11 nucleotides between them. Alignment of these two variants with the two most similar sequences of *Hepatozoon* from the GenBank database is given in Additional file 2. Phylogenetic analysis revealed that our *Hepatoozon* sequences grouped together with genotypes/species of *Hepatozoon* derived from other species of rodents from different parts of the world [i.e. *A.olivaceus, A. sanborni, Bandicota indica, Jaculus* spp.*, Sciurus vulgaris, Clethrionomys* (*Myodes*) glareolus] and from some species of reptiles, but were distant from *Hepatozoon* genotypes/species found in carnivores (i.e. *H. felis, H. americanum, H. ursi*) (Fig. 1).

#### *Trypanosoma* sp.

Forty - five *Trypanosoma* isolates were obtained and genotyped: 13 isolates from 2004, 10 isolates from 2008 and 22 isolates from 2012. All isolates were derived from *A. dimidiatus*, mostly from just two wadis where this parasite was found during the study period: 15 isolates from W. Gharaba and 29 isolates from W. Tlah. Additionally, one isolate from W. Gebal was obtained and genotyped. Isolates were genotyped by the amplification and sequencing of a 520-bp fragment of the 18S rRNA gene. Two genetic variants of *Trypanosoma* were identified, variant A and B. Variant A was widespread, and identified in 44 *A. dimidiatus* from W. Gebal, Gharaba and Tlah (2004, 2008 and 2012). One representative sequence of variant A was deposited in the GenBank database under the accession number KT337473. Variant B was identified only in one isolate from *A. dimidiatus* from W. Tlah in 2012. The sequence of this variant was deposited in the GenBank database under the accession number KT337474.

A BLAST search in the GenBank database revealed, that both variants A and B showed the highest sequence homology to a *Trypanosoma* sp. isolate from *A. dimidiatus* from one of our own earlier expeditions to Egypt (100 %; HQ324793) (direct submission). The next closet matches (95.94 %) of variant A were *Trypanosoma* sp. from Anderson’s red-backed vole, *Eothenomys andersoni,* from Japan (AB242276) and *T. microti* from the field vole, *Microtus agrestis,* from the UK (AJ009158). Variant B showed the next highest homology (97.39 %) to *Trypanosoma* sp. B08-471 from a squirrel flea, *Ceratophyllus* (*Monopsyllus*) *sciurorum* from the Czech Republic (KF054111) and to *T. microti* from *M. agrestis* from the UK (AJ009158).

**Fig. 2:**
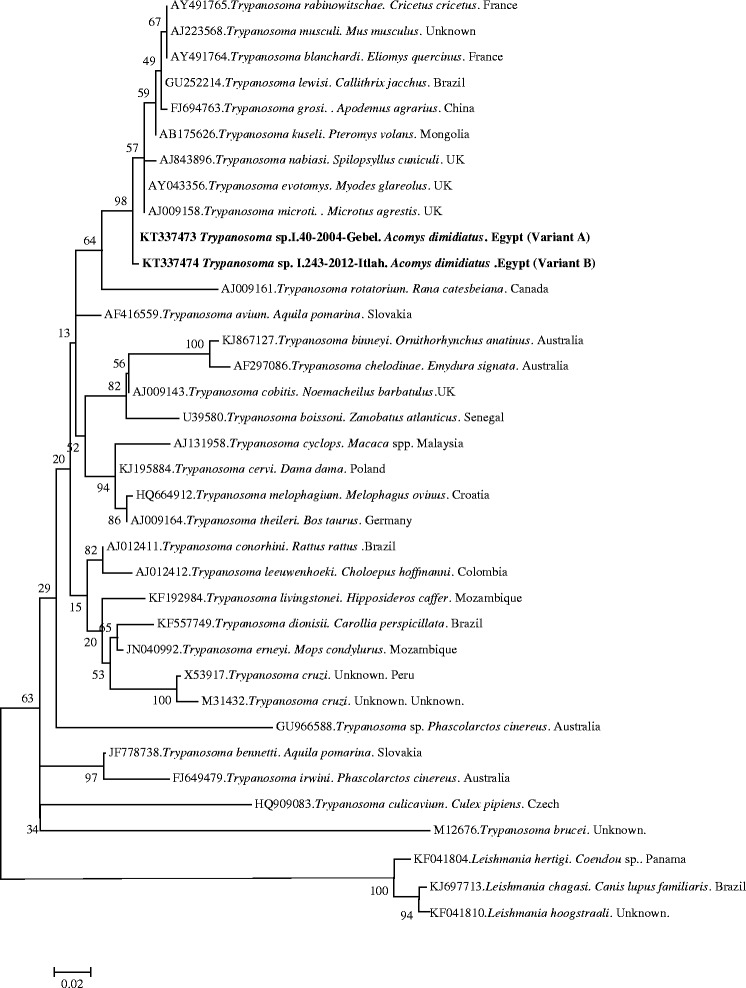
The evolutionary history of *Trypanosoma*, based on the fragment of the 18S rRNA gene, was inferred using the Maximum Likelihood method and a Tamura 3-parameter (I + G). The percentage of replicate trees in which the associated taxa clustered together in the bootstrap test (1000 replicates) are shown next to the branches. The analysis involved 36 nucleotide sequences. All positions containing gaps and missing data were eliminated. The nucleotide sequence of *Leishmania hertigi, L. chagasi* and *L. hoogstraali* were used as outgroups. Evolutionary analyses were conducted in MEGA6.0

Alignment of our two *Trypanosoma* variants revealed a difference of 7 nucleotides between them. Alignment of these two variants with the most similar sequences of *Trypanosoma* from the GenBank database is presented in Additional file 3. Phylogenetic analysis revealed that our *Trypanosoma* sequences grouped together with species of *Trypanosoma* derived from other species of rodents from different parts of the world (*T. blanchardi, T. evotomys, T. grosi, T. kuseli, T. lewisi, T. microti, T. musculi*) and were distant from the key pathogenic species, *T. brucei* or *T. cruzi* (Fig. 2).

### Environmental study in Sinai in the period 2000–2012

#### *Acomys dimidiatus *

A total of 857 individual spiny mice were caught over the four surveys (Table 2) and blood samples of sufficient quality to assess microscopically were obtained from 835 (Table 3). Values for the trapping effort and the resulting success in terms of mice caught/100 trap hours (th), and number of mice as a percentage of traps deployed in the field are summarized by wadi and year of survey in Table 2. Analysis of these data by the Kruskal-Wallis test with either YEAR or SITE as the explanatory factor on each of the variables listed in turn, revealed that the only significant effect was that of YEAR on the number of trap hours (*χ*^2^_3_ = 11.5, *P* = 0.009). As can be seen in Table 2, the number of trap hours was larger in 2008 and 2012 compared with the two earlier years.

**Table 2:** Trapping effort and relative population size of *A. dimidiatus*

		Trap Hours	No. of *A. dimidiatus*	Mice/100 th^a^	Trap success (%)^b^
Arbaein	2000	2723	63	2.31	32.0
	2004	3265	43	1.32	16.8
	2008	3714	69	1.86	25.3
	2012	3918	67	1.71	23.4
	Combined	13620	242	1.78	23.9
Gebel	2000	1838	32	1.74	21.3
	2004	2112	43	2.04	27.4
	2008	3831	43	1.12	16.4
	2012	3675	47	1.28	17.2
	Combined	11456	165	1.44	19.6
Gharaba	2000	2136	28	1.31	16.5
	2004	2913	61	2.09	29.2
	2008	4314	54	1.25	16.1
	2012	3989	52	1.30	17.2
	Combined	13352	195	1.46	19.2
Tlah	2000	2199	46	2.09	27.7
	2004	2117	70	3.31	45.2
	2008	5344	80	1.50	20.1
	2012	3988	59	1.48	19.9
	Combined	13648	255	1.87	25.1
Combined	2000	8896	169	1.90	24.7
	2004	10407	217	2.09	27.9
	2008	17203	246	1.43	19.3
	2012	15570	225	1.45	19.4
	Grand total/average	52076	857	1.65	22.0

^a^No of animals caught per 100 trap hours (th)

^b^Trap success = percentage of traps occupied by a mouse after overnight deployment of traps in the study sites

**Table 3:** Numbers of *Acomys dimidiatus* examined by site, year, host age and sex

			Age class			Totals	
Site	Year	Sex	Class1	Class2	Class3	Row	Site and year
El Arbaein	2000	Males	6	15	7	28	
		Females	11	8	11	30	58
	2004	Males	4	5	12	21	
		Females	7	6	9	22	43
	2008	Males	3	10	17	30	
		Females	11	9	16	36	66
	2012	Males	8	3	18	29	
		Females	9	11	15	35	64
	All years	Total males	21	33	54	108	
		Total females	38	34	51	123	
		Total combined sexes	59	66	105	231	
Gebal	2000	Males	3	7	2	12	
		Females	3	5	8	16	28
	2004	Males	4	2	8	14	
		Females	8	1	20	29	43
	2008	Males	5	2	12	19	
		Females	4	5	15	24	43
	2012	Males	4	7	10	21	
		Females	7	1	16	24	45
	All years	Total males	16	18	32	66	
		Total females	22	12	59	93	
		Total combined sexes	38	30	91	159	
Gharaba	2000	Males	1	5	5	11	
		Females	2	6	9	17	28
	2004	Males	5	8	12	25	
		Females	6	10	19	35	60
	2008	Males	7	3	14	24	
		Females	8	5	15	28	52
	2012	Males	1	4	14	19	
		Females	6	8	19	33	52
	All years	Total males	14	20	45	79	
		Total females	22	29	62	113	
		Total combined sexes	36	49	107	192	
Tlah	2000	Males	4	9	11	24	
		Females	4	5	13	22	46
	2004	Males	2	15	17	34	
		Females	7	8	21	36	70
	2008	Males	13	12	14	39	
		Females	12	12	16	40	79
	2012	Males	4	7	10	21	
		Females	11	8	18	37	58
	All years	Total males	23	43	52	118	
		Total females	34	33	68	135	
		Total combined sexes	57	76	120	253	
Total by year	2000	Males	14	36	25	75	
		Females	20	24	41	85	
		Both sexes	34	60	66	160	
	2004	Males	15	30	49	94	
		Females	28	25	69	122	
		Both sexes	43	55	118	216	
	2008	Males	28	27	57	112	
		Females	35	31	62	128	
		Both sexes	63	58	119	240	
	2012	Males	17	21	52	90	
		Females	33	28	68	129	
		Both sexes	50	49	120	219	
Total by sex		Males	74	114	183	371	
		Females	116	108	240	464	
		Both sexes	190	222	423	835	

The structure and the sample sizes of each subset of the host population that was assessed for haemoparasites, is shown in Table 3 by site and year of study, host sex and age. The numbers of mice sampled differed significantly between the wadis (*χ*^2^_3_ = 25.4, *P* < 0.001), with most from W. Tlah and the least from W. Gebal. The distribution of mice among age classes also varied significantly between the sexes (SEX × AGE, *χ*^2^_2_ = 6.8, *P* = 0.033) and between the four surveys (YEAR × AGE, *χ*^2^_6_ = 15.2, *P* = 0.019), and these effects are taken into consideration in further analyses.

### Haemoparasites - all species combined

**Fig. 3:**
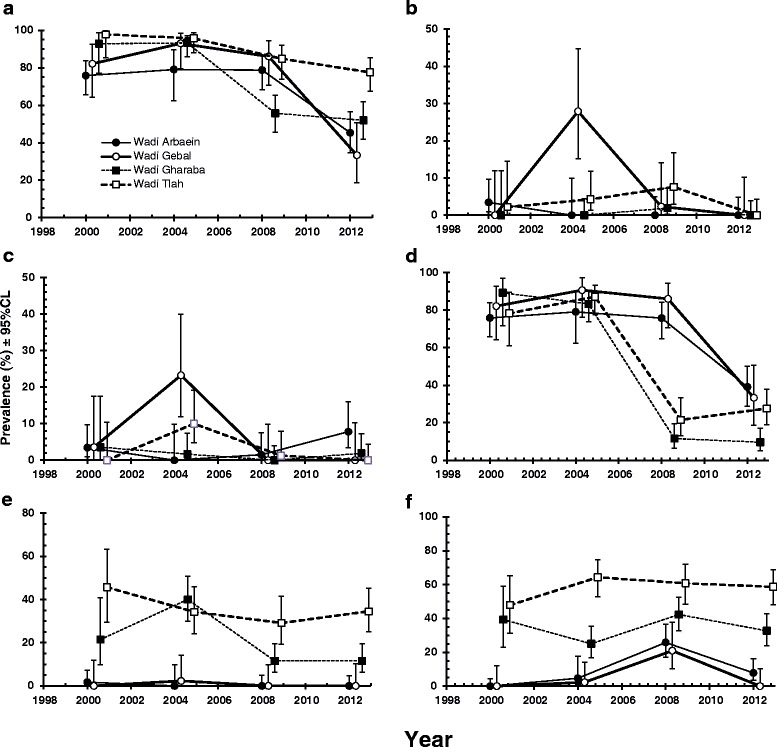
Prevalence of haemoparasites recorded in spiny mice at the four study sites in the Sinai between 2000 and 2012. **a**, all haemoparasites, combined; **b**, *Babesia* sp.; **c**, *Bartonella* sp.; **d**, *Haemobartonella* sp.; **e**, *Trypanosoma acomys*; **f**, *Hepatozoon* sp. The key to symbols used for the four study sites are given in panel A

Overall 76.2 % (72.61–79.42) of the 835 spiny mice carried at least one of the five haemoparasite genera recorded in the study. Prevalence varied significantly between the sites (Table 4; SITE × INFECTION, *χ*^2^_3_ = 34.5, *P* < 0.001) with the highest overall prevalence in W. Tlah and the lowest in W. El Arbaein, and it changed significantly with time (Table 4; YEAR × INFECTION, *χ*^2^_3_ = 99.1, *P* < 0.001), although the pattern of change of prevalence with time varied between the sites (Fig. 3a; YEAR × SITE × INFECTION, *χ*^2^_9_ = 29.7, *P* < 0.001). In the first two surveys there was little change in prevalence, but in 2008 prevalence began to drift downwards, especially in W. Gharaba and by 2012, prevalence had fallen in all four sites relative to 2000, although the least change was observed in mice from W. Tlah. Age on its own was just the wrong side of significance (AGE × INFECTION *χ*^2^_2_ = 5.9, *P* = 0.051) and as can be seen in Table 4, prevalence was lowest among the young age class but only 9 % higher in the intermediate age class and slightly lower among the oldest mice, so overall little change with increasing host age. However, the age of hosts featured also in two interactions, one with year of survey (YEAR × AGE × INFECTION, *χ*^2^_6_ = 13.5, *P* = 0.036) and one with location of sampling (SITE × AGE × INFECTION *χ*^2^_6_ = 19.0, *P* = 0.004), which we did not explore further. There was no significant difference in prevalence between the two sexes and SEX did not figure in any of the interactions that were detected.

**Table 4:** Prevalence of haemoparasites by year, site, host sex and age class

	Haemoparasites	*Babesia* spp.	*Bartonella* spp.	*Haemobartonella* spp.	*Trypanosoma acomys*	*Hepatozoon* spp.
Year						
2000	**86.3** (79.11–91.34)	**1.9** (0.44–6.20)	**2.5** (0.76–7.00)	**80.0** (72.16–86.30)	**17.5** (11.68–25.22)	**20.6** (14.26–28.47)
2004	**91.2** (88.25–93.47)	**6.9** (4.91–9.61)	**8.3** (6.12–11.24)	**85.2** (81.69–88.17)	**22.7** (19.08–26.70)	**29.2** (25.18–33.48)
2008	**77.1** (72.86–80.85)	**3.3** (1.97–5.48)	**0.8** (0.31–2.25)	**45.8** (41.09–50.58)	**12.1** (9.28–15.53)	**40.0** (35.42–44.74)
2012	**53.0** (48.40–57.53)	**0** (0–0.82)	**2.7** (1.56–4.65)	**27.9** (23.92–32.10)	**11.9** (9.18–15.16)	**25.6** (21.78–29.76)
Site						
Arbaein	**68.8** (64.36–72.98)	**0.9** (0.33–2.27)	3.5 (2.08–5.59)	**66.2** (61.67–70.55)	**0.4** (0.16–1.57)	**10.4** (7.80–13.61)
Gebal	**72.3** (63.96–79.50)	**8.2** (4.38–14.45)	6.9 (3.61–12.75)	**71.7** (63.24–78.90)	**0.6** (0.07–4.02)	**6.3** (3.17–11.88)
Gharaba	**71.9** (62.51–79.80)	**0.5** (0.04–4.43)	1.6 (0.25–6.27)	**44.8** (35.47–54.56)	**21.9** (14.92–30.98)	**33.9** (25.33–43.58)
Tlah	**88.5** (85.06–91.34)	**4.0** (2.41–6.30)	3.2 (1.81–5.34)	**51.4** (46.52–56.24)	**34.8** (30.27–39.55)	**58.9** (54.04–63.63)
Sex						
Males	76.0 (70.62–80.73)	4.0 (2.21–7.04)	3.0 (1.49–5.70)	56.9 (51.00–62.58)	15.4 (11.55–20.08)	28.3 (23.29–33.90)
Females	76.3 (70.13–81.59)	2.4 (0.97–5.43)	4.1 (2.04–7.60)	58.6 (51.94–65.09)	16.2 (11.75–21.64)	30.8 (25.03–37.24)
Age						
Class 1	69.5 (59.84–77.81)	**6.3** (2.95–12.62)	4.2 (1.56–9.94)	60.0 (50.30–69.17)	**15.3** (9.32–23.40)	**9.5** (5.06–16.61)
Class 2	78.8 (74.85–82.40)	**0.9** (0.35–2.28)	3.2 (1.86–5.17)	57.7 (53.08–62.14)	**26.6** (22.69–30.84)	**36.9** (32.56–41.51)
Class 3	77.8 (72.12–82.65)	**2.8** (1.33–5.81)	3.5 (1.77–6.70)	57.0 (50.64–63.11)	**10.4** (7.03–14.92)	**35.0** (29.14–41.28)

Significant main effects are highlighted in bold

**Fig. 4:**
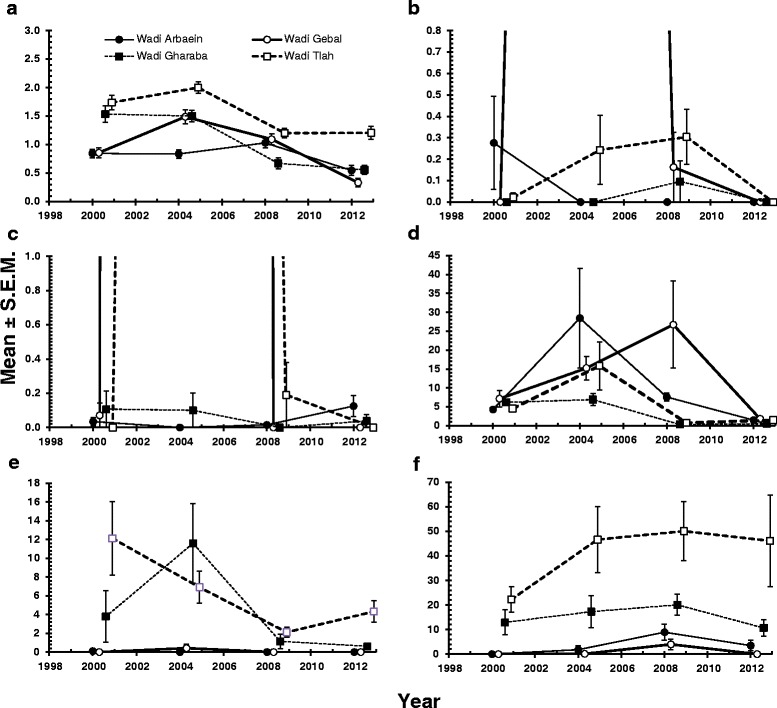
Abundance of haemoparasites recorded in spiny mice at the four study sites in the Sinai between 2000 and 2012. **a**, species richness; **b**, *Babesia* sp.; **c**, *Bartonella* sp.; **d**, *Haemobartonella* sp.; **e**, *Trypanosoma acomys*; **f**, *Hepatozoon* sp. In b, the abundance of *Babesia* sp. in mice from Wadi Gebal in 2014 was 13.7 ± 11.60, and in c the abundance of *Bartonella* sp. in mice from wadis Gebal and Tlah was 257.98 ± 191.37 and 31.9 ± 28.62, respectively. The key to symbols used for the four study sites are given in panel A

**Fig. 5:**
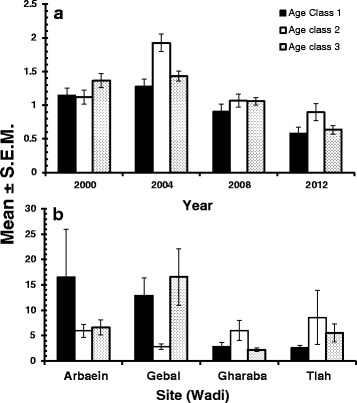
Variation in haemoparasites in spiny mice sampled by age class. **a**, species richness by year of survey; **b**, abundance of *Haemobartonella* sp. by site

The mean species richness was 1.2 ± 0.03. The best fit model was one with a Gaussian error structure (YEAR + SITE + AGE + YEAR x SITE + YEAR x AGE on species richness, adjusted *R*^2^ = 0.31). The data in Table 5 show that species richness peaked in 2004 and dropped by more than 50 % to 2012 (main effect of YEAR, *F*_3,826_ = 45.9, *P* < 0.0001), was highest among mice from W. Tlah (main effect of SITE, *F*_3,826_ = 38.1, *P* < 0.0001) and peaked in age class 2 mice (main effect of AGE, *F*_2,826_ = 5.5, *P* = 0.004). While species richness was consistently highest among mice from W. Tlah, there was significant variation in the rank order of species richness among mice for the other wadis from survey to survey (Fig. 4a; 2-way interaction YEAR × SITE, *F*_9,820_ = 7.5, *P* < 0.0001), as illustrated for example among mice from W. Gharaba for which species richness was second highest in 2000 and 2004, but lowest in 2008. The profile of species richness across the three age classes also varied between surveys (Fig. 5a; YEAR × AGE, *F*_6, 817_ = 3.1, *P* = 0.006). In three surveys (2004, 2008 and 2012), species richness was highest among age class 2 mice, but the pattern was different in 2000 with a peak among the oldest animals.

**Table 5:** Abundance of haemoparasites by year, site, host sex and age class

	Species richness		*Babesia* sp.		*Bartonella* sp.		*Haemobartonella* sp.		*Trypanosoma acomys*		*Hepatozoon* sp.	
	Mean ± S.E.M.		Mean ± S.E.M.		Mean ± S.E.M.		Mean ± S.E.M.		Mean ± S.E.M.		Mean ± S.E.M.	
Year												
2000	**1.23**	0.063	**0.11**	0.079	**0.04**	0.024	**5.17**	0.502	**4.19**	1.279	**8.65**	1.889
2004	**1.53**	0.058	**2.81**	2.318	**61.72**	39.432	**15.73**	3.435	**5.55**	1.337	**20.29**	4.884
2008	**1.02**	0.047	**0.15**	0.056	**0.07**	0.063	**7.18**	2.159	**0.94**	0.249	**23.98**	4.334
2012	**0.68**	0.050	**0**	0	**0.05**	0.020	**1.30**	0.211	**1.29**	0.330	**15.75**	5.146
Site												
Arbaein	**0.81**	0.042	**0.07**	0.055	**0.05**	0.019	**8.93**	2.543	**0.03**	0.026	**3.84**	1.167
Gebal	**0.94**	0.059	**3.75**	3.147	**69.78**	52.114	**13.09**	3.319	**0.11**	0.113	**1.10**	0.583
Gharaba	**1.03**	0.060	**0.03**	0.026	**0.06**	0.036	**3.28**	0.571	**4.65**	1.438	**15.59**	2.629
Tlah	**1.52**	0.056	**0.17**	0.06	**8.89**	7.929	**5.76**	1.797	**5.77**	0.925	**43.14**	6.835
Sex												
Males	1.08	0.043	0.25	0.073	5.72	5.394	7.68	1.820	1.55	0.262	16.21	2.741
Females	1.12	0.039	1.22	1.079	24.23	17.889	7.29	1.352	3.88	0.766	19.30	3.433
Age												
Class 1	**0.95**	0.057	**0.38**	0.122	63.32	44.54	8.98	3.046	**1.66**	0.419	**2.25**	0.772
Class 2	**1.26**	0.062	**0.13**	0.097	1.09	0.757	6.44	1.897	**3.23**	0.531	**29.32**	6.697
Class 3	**1.09**	0.039	**1.32**	1.183	2.58	2.365	7.32	1.375	**3.18**	0.806	**19.00**	2.664

Significant main effects are highlighted in bold

#### *Babesia* spp.

The overall prevalence of *Babesia* spp. was just 3.1 % (1.97–4.81). Prevalence varied from 6.9 % in 2004, to zero in 2012, and the difference between the surveys was significant (Table 4; YEAR × INFECTION, *χ*^2^_3_ = 22.7, *P* < 0.001), as was that also between sites (Table 4; SITE × INFECTION *χ*^2^_3_ = 21.9, P < 0.001). Prevalence was highest in W. Gebal and lowest in W. Gharaba, where just one of the 192 spiny mice examined was found to be infected. However, there was also a significant interaction (YEAR × SITE × INFECTION, *χ*^2^_9_ = 22.7, *P* = 0.007) and this is illustrated in Fig. 3b. Infections were sporadic in three of the wadis, recorded in only 1 or 2 of the 4 surveys, except in W. Tlah where *Babesia* was detected in three of the four surveys. There was also a marked peak in prevalence in 2004 in mice from W. Gebal, where otherwise in 2000 and 2012 no infections were detected. Prevalence was higher among the youngest age class (Table 4; AGE × INFECTION *χ*^2^_2_ = 10.8, *P* = 0.004) compared with the older age classes, but there was no significant difference in prevalence between the sexes.

With just 26 out of 835 animals infected with *Babesia* sp., the overall mean abundance was 0.79 ± 0.600 IN/200FV. Quantitative analysis was problematic with parametric models with Gaussian errors on raw and log transformed data failing to generate acceptable distributions of residuals and to those with negative binomial errors failing to converge (See Methods). Therefore, only the main effects were tested using non-parametric tests, with much the same outputs as those for prevalence (Kruskal-Wallis test for the effects of YEAR, *χ*^2^_3_ = 18.6, *P* < 0.001; SITE, *χ*^2^_3_ = 22.5, *P* < 0.001 and AGE, *χ*^2^_2_ 10.1, *P* = 0.006). There was no significant difference between the sexes (Mann-Whitney *U* test *Z* = -1.37, *P* = 0.17). The mean values are given in Table 5 and the SITE x YEAR effect in Fig. 4b. Mostly mean abundance was less than 0.4 IC/200FV, with the exception of W. Gebal when in 2004, twelve mice were infected, and one with 500 IC/200 FV, generating a mean for this site of 13.7 ± 11.60 for that year.

#### *Bartonella* spp.

The prevalence of *Bartonella* spp. was also very low (3.6 %, 2.36–5.38). Prevalence varied significantly between years (Table 4; YEAR × INFECTION *χ*^2^_3_ = 19.0, *P* < 0.001) with a peak in 2004, mostly accounted for by high values among mice from Wadis Gebal and Tlah. Prevalence also rose in W. El Arbaein in 2012, but otherwise values were very low. There was no independent effect of SITE, but the significant SITE × YEAR × INFECTION interaction (*χ*^2^_9_ = 28.7, *P* = 0.001) is illustrated in Fig. 3c. There were no independent effects of host sex or age, but there was a complex interaction involving these factors (SEX × SITE × AGE × INFECTION, *χ*^2^_6_ = 19.6, *P* = 0.003) which we did not explore further.

As with *Babesia*, quantitative analysis of abundance of *Bartonella* sp. was problematic because so few animals were infected (30/835). The overall mean abundance was 16.0 ± 10.23 IC/200FV, but this relatively high value was largely attributable to young mice from Wadis Gebal (mean = 258.0 ± 191.4 with values exceeding 1000 IC/200FV in three mice, maximum =8000) and Tlah (mean = 31.9 ± 28.6; maximum = 2000) in 2004 (Fig. 4c and Table 5). In marked contrast, in the remaining surveys the mean abundance values did not exceed 0.3 IC/200 FV in the other wadis. Analysis was by non-parametric tests which showed that there was a hugely significant difference between surveys (Table 5; Kruskal-Wallis test with YEAR, *χ*^2^_3_ = 20.9, *P* < 0.001) and a weaker effect of SITE (*χ*^2^_3_ = 7.8, *P* = 0.05). There was no significant difference between the sexes or between the age classes.

#### *Haemobartonella* spp.

The overall prevalence of *Haemobartonella* spp. was 57.8 % (53.88–61.73). However, as can be seen in Table 4 and Fig. 3d, initially in the first two surveys prevalence was higher exceeding 75 % in four wadis, but then prevalence fell markedly in two wadis in 2008 (Gharaba and Tlah), and the other two also by 2012 (YEAR × INFECTION, *χ*^2^_3_ = 205.4, *P* < 0.001). The difference in prevalence between wadis was also significant (Table 4; SITE × INFECTION, *χ*^2^_3_ = 37.4, *P* < 0.001), with the highest overall value in W. Gebal and the lowest in W. Gharaba. Prevalence of this species was not affected by either host age or sex, but there was a significant interaction between all four factors and INFECTION (*χ*^2^_18_ = 35.9, *P* = 0.007), suggesting some differences between age classes and the two sexes in particular data subsets (i.e. in particular years and sites) which we did not explore further.

The mean abundance for *Haemobartonella* sp. was 7.5 ± 1.10 IC/200FV. The best-fit model was a GLM with negative binomial errors (YEAR + SITE + AGE + YEAR x SITE+ SITE x AGE). There were highly significant main effects of YEAR (*LR*_3,826_ = 182.1, *P* < 0.0001) and SITE (*LR*_3,826_ = 76.5, P < 0.0001) and these are summarised in Table 5. Abundance peaked in 2004 and was lowest in 2012, and peak abundance was detected in mice from W. Gebal, whilst the lowest value was from those from W. Gharaba. The highly significant interaction between SITE and YEAR (*LR*_9,811_ = 120.9, *P* < 0.0001) is illustrated in Fig. 4d. This shows the dynamic changes that occurred between surveys, with peak abundance among mice from W. Gebal in three surveys (2000, 2008 and 2012) but not in 2004 when peak abundance was among mice from W. El Arbaein. There was no main effect of host age (*LR*_2,826_ = 0.78, *P* = 0.96), but there was a significant interaction between SITE and AGE (*LR*_6,811_ = 19.5, *P* = 0.0034) which is illustrated in Fig. 5b. As can be seen, the age-prevalence profiles were quite different among mice from each of the 4 wadis, although there was some similarity between those from Wadis Gharaba and Tlah where overall prevalence was lowest. Peak prevalence was observed in age class 1 mice in Wadi El Arbaein, in age class 3 in Wadi Gebal and in age class 2 in Wadis Gharbara and Tlah. An alternative model based on log-transformed abundance and Gaussian errors generated much the same output (*R*^2^ = 0.34).

#### *Trypanosoma acomys*

The overall prevalence of *T. acomys* was 15.8 % (13.09–18.92). Although prevalence changed significantly over the four surveys, peaking in 2004 (Table 4; YEAR × INFECTION, *χ*^2^_3_ = 12.5, *P* = 0.006), there was a huge difference between the wadis (SITE × INFECTION, *χ*^2^_3_ = 175.3, *P* < 0.001), with this species being largely confined to two of the four study sites in all four surveys (Gharaba and Tlah; Fig. 3e). There was no difference in prevalence between the two sexes, but there was a significant difference between age classes (AGE × INFECTION, *χ*^2^_2_ = 26.8, *P* < 0.001). Prevalence was highest in age class 2 (Table 4), and then dropped by more than 50 % in the oldest age class. There were no significant confounding interactions in this case.

**Fig. 6:**
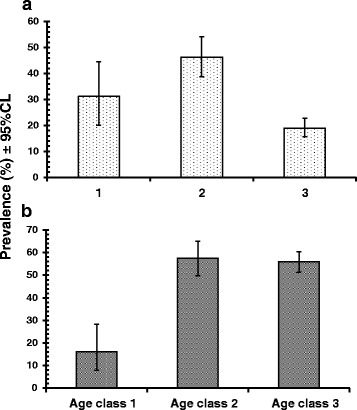
Prevalence of *T. acomys* **a** and *Hepatozoon* sp. **b** in mice of three age classes, restricted to animals from Wadis Gharaba and Tlah

Since this parasite was largely confined to just two of the four wadis, we recalculated the effect of age on prevalence restricting the data to mice from wadis Gharba and Gebal, and this is shown in Fig. 6a. Prevalence in age class 2 spiny mice was now 46.4 % (38.75–54.16), falling to 18.9 % (15.57–22.85) among the oldest mice, a reduction of 59.2 %.

The mean abundance of *T. acomys* was 2.85 ± 0.443. Parametric models based on raw data with Gaussian or negative binomial errors, and log_10_(X + 1) transformed data with Gaussian errors all failed to generate acceptable distributions of residuals. However analysis by 1-way non-parametric tests identified a marked effect of SITE (Table 5; Kruskal-Wallis test, χ^2^_3_ = 141.5, *P* < 0.001) mean abundance of trypanosomes being higher in mice from W. Gharaba and Tlah. There were also significant changes in abundance between the 4 surveys (Kruskal-Wallis test on effect of YEAR χ^2^_3_ = 16.1, *P* = 0.001) with mean abundance higher in the first two surveys compared with the latter two. Abundance also increased with host age, especially between the youngest mice and age class 2, and then fell marginally in the oldest mice (Kruskal-Wallis test on effect of AGE χ^2^_2_ = 26.8, *P* < 0.001). There was no significant difference in abundance between the sexes. Changes of abundance over time in the 4 wadis are illustrated in Fig. 4e, where it can be seen clearly that mean abundance was consistently higher among mice from Wadis Gharaba and Tlah compare to those from Wadis El Arbaein and Gebal.

#### *Hepatozoon* sp.

The prevalence of this species was 29.7 % (26.17–33.47). As with *T. acomys*, *Hepatozoon* sp. was largely confined to the same two wadis (Gharaba and Tlah), and not surprisingly there was a huge SITE effect (SITE × INFECTION, *χ*^2^_3_ = 198.6, *P* < 0.001). Prevalence also varied between the surveys (YEAR × INFECTION, *χ*^2^_3_ = 20.2, *P* < 0.001) and differently among the mice from the four wadis (Fig. 3f; SITE × YEAR × INFECTION, *χ*^2^_9_ = 36.1, *P* < 0.001). There was no effect of host sex, but age affected prevalence significantly (Table 4; *χ*^2^_2_ = 56.7, *P* < 0.001), and the precise effect of age on prevalence varied between the surveys (Not illustrated, AGE × YEAR × INFECTION, *χ*^2^_6_ = 17.7, *P* = 0.007). Very few of the youngest mice were infected, but then prevalence increased and stabilized in mature and older individuals some 3–4 times higher (Table 4). However, since this species, like *T. acomys*, was largely confined to mice from Wadis Gharaba and Tlah, the analysis was repeated excluding individuals from the other two wadis and this is illustrated in Fig. 6b. Prevalence was still very low among the youngest mice (16.1 % [7.95–28.36]), but rose to over 55 % among age classes 2 and 3.

The mean abundance of this species was 17.9 ± 2.26. Parametric models based on raw data with Gaussian or negative binomial errors, and log_10_(X + 1) transformed data with Gaussian errors all failed to generate acceptable distributions of residuals. Analysis by 1-way non-parametric tests identified a marked effect of SITE (Table 5; Kruskal-Wallis test, χ^2^_3_ = 190.8, *P* < 0.001), mean abundance of *Hepatozoon* sp. being highest in W. Tlah and lowest in W. Gebal. There were also significant changes in abundance between the 4 surveys with peak abundance detected in 2008 (Kruskal-Wallis test on effect of YEAR χ^2^_3_ = 21.2, *P* = 0.001). As with *T. acomys* abundance was lowest in the youngest mice, higher among age class 2 before falling in age class 3 (Kruskal-Wallis test on effect of AGE χ^2^_2_ = 51.1, *P* < 0.001). There was no significant difference in abundance between the sexes. Changes of abundance over time in the four wadis are illustrated in Fig. 4f, where the consistently high abundance among mice from W. Tlah can be seen. This is followed by a consistent intermediate level of abundance among mice from W. Gharaba and much lower abundance among animals from the remaining two wadis, although there was a small peak in abundance in 2008 among mice from the latter two sites.

## Discussion

### Molecular identification of parasites

Molecular techniques were used for the identification of parasite species/genotype and for a preliminary study of the diversity of parasites among the four semi-isolated populations of spiny mice. Analyses of the sequences that we obtained did not allow exact identification of species (no 100 % sequence homology was found in the GenBank database) of *Bartonella, Trypanosoma* or *Hepatozoon* as to-date, excepting our own depositions, no data on the haemoparasites specific to *A. dimidiatus* have been deposited. The closest match was for *Bartonella*, as our sequence was >99 % similar to *B. acomydis*, the species described from the golden spiny mouse, *A. russatus* [35]. As *Bartonella* species/genotypes show some host-specificity, further study on the *Bartonella* from *A. dimidiatus* is needed to clarify with certainty the identity of the parasite in the spiny mice in our study sites. Interestingly, in our study more *Bartonella*-positive mice were detected by conventional microscopy of blood smears, but only one PCR product was obtained with the primers that we used. In Inoue et al. [35], the study referred to above, the overall prevalence of *Bartonella* in *A. dimidiatus* imported from Egypt to Japan was 9.7 %, relatively low as in our study, but in three isolates *B. elizabethae* was identified (100 % homology with GenBank reference sequences), a species that is pathogenic for humans. Therefore, it is still possible that other species/strains of *Bartonella* may be found in the future in spiny mice from our S. Sinai study sites.

There is only one species of *Trypanosoma* described from spiny mice, *T. acomys* [37, 38], but no reference sequences for 18S rDNA of this species are available in GenBank. However, almost no diversity was observed in the 45 *Trypanosoma* sequences that we compared and this supports the identification of our isolates as the host-specific *T. acomys*. Interestingly, one of our sequences was different (variant B), but still most closely related to our dominant variant- variant A of *T. acomys. T. acomys* was found in mice from two quite distant wadis (W. Tlah and W. Gharaba) which despite the distance between them are connected, W. Gharaba being located at the extreme north end of the continuous valley system leading away from St. Katherine [9], and there was no detectable diversity between the isolates from these two wadis.

The genotyping of *Hepatozoon* isolates from the two *Acomys* species revealed the presence of two variants, A and B, and neither of these could be identified to species level through comparison with data in GenBank (at best homology was 96–97 % with known *Hepatozoon* species). Only one species of *Hepatozoon* has been described from *Acomys* spp. - *H. acomys* (Mohammed and Saound 1972, following [39]) and generally the systematics of this genus are still poorly developed [36, 39]. The majority of identified *Hepatozoon* infections from different groups of animals, including amphibians, reptiles and mammals, are reported simply as ‘*Hepatozoon* sp.’ However, even among the conserved 18S rDNA sequences of those *Hepatozoon* spp. isolates that have been deposited, there are significant differences (Fig. 1) and there is an urgent need to name the different genotypes of *Hepatozoon* that are known to be associated with certain host species. Only one detailed description of *Hepatozoon* from Egyptian rodents is currently available- *H. balfouri* in *J. jaculus* and *J. orientalis* [40]. However, the gamonts of this species were found only in erythrocytes, and never in leucocytes, in contrast to the *Hepatoozon* that we observed in *A. dimidiatus*. Moreover, a recent study of *Hepatozoon* from jerboas revealed sequences that were quite different to our own isolates (Fig. 1; [36]), supporting the existence of a different *Hepatozoon* species in *A. dimidiatus*. Interestingly, our variant B of *Hepatozoon* was found only in one of the two *Acomys* species, *A. russatus*, which is consistent with the idea of host-specificity among parasites in this genus.

### Long-term ecological study

Our data show that the haemoparasite communities varied markedly between the four subpopulations of spiny mice living in isolated wadis and displayed long-term trends, most likely associated with the increasing aridity of the environment during the years of our surveys. The haemoparasite communities of *A. dimidiatus* were dynamic, with only some species showing stability across the 12-year-long period. As predicted, external factors (site and year of study) had a much greater influence on the haemoparasite community than the intrinsic factors that we took into account in our analyses (host age or sex).

Long-term dynamic changes of haemoparasites were observed in prevalence, abundance and in mean species richness, as reflected in the spatio-temporal patterns/trends illustrated in Figs. 3 and 4 (the year x site interactions). While the prevalence of haemoparasites was relatively high and stable during the first two surveys, in 2000 and 2004, there was a 50 % reduction by the last survey in 2012, and this pattern of declining prevalence was observed clearly among the spiny mice in 3 of the 4 wadis in our study (W. El Arbaein, W. Gebal, W. Gharaba), with only slightly lower values for mice from W. Tlah. Mean species richness was the highest in 2004 and then decreased by more than 50 % to 2012. Both of these two parameters (prevalence and mean species richness) were significantly reduced in 2008 and then in 2012, compared with the earlier surveys. The fall in value of both parameters may have two non-mutually exclusive explanations– an exceptionally high prevalence of two ‘rare’ pathogens, *Babesia* and *Bartonella* sp. in 2004, exceeding 20–30 % in W. Gebal, and the marked decrease in *Haemobartonella* sp., in 2008 in W. Tlah and Gharaba, and then in 2012 in W. El Arbaein and Gebal. Although overall prevalence of *T. acomys* was generally stable over the 12-year-long period, the greatest stability was observed in W. Tlah while in W. Gharaba prevalence of *Trypanosoma* followed the general trend in the prevalence of other haemoparasites – a significant, albeit relatively small, reduction during last two surveys, contributing to an overall drop in haemoparasite prevalence and mean species richness. Interestingly, even for this ‘stable’ parasite, the abundance and intensity of infection were much lower in 2008 and 2012 in both W. Tlah and Gharaba, compared to the first two surveys. An opposite trend was found for *H. acomys* – the only parasite species displaying an increase in prevalence in 2008 and a spread to new sites.

This fall in the value of three parameters must reflect a drop in transmission of these vector-borne parasites. There have been severe fluctuations in the weather in the Sinai over the past decade (2002–2010). Since 2002 there has been a severe drought with very little (< 50 mm) or no rain every year until March 2010 and therefore one underlying explanation could be the marked decrease in water availability, first noted during the expedition in 2008. This was the first expedition during which we observed a lack of water pipes that are usually employed to deliver water to Bedouins’ gardens situated at higher altitudes in the wadis, the lack of water in wells located in the wadis, the abandonment of several gardens in W. Gebal and Gharaba, resulting not only in a lack of ground-cover plants and vegetables but also in desiccation of trees. This drought was broken in May 2010 when there was heavy rainfall. 2011 was again extraordinarily wet, with heavy rainfall and snow in the winter and spring, while 2012 had very little rainfall and was colder than normal. Although almost the wettest place in Egypt (second only to the Mediterranean northwestern coast), according to the best (patchy) data we have, the mountains of South Sinai received only an average of 42.5 mm per year precipitation between 1970 and 1994, and substantially less (15.5 mm) between 2001 and 2009 (data courtesy of the St Katherine Protectorate Management Unit) [28]. These weather conditions have probably caused the marked changes recorded in a parallel long-term study on the Sinai thyme (*Thymus decussatus*) population. Between 2002 and 2010, the number of thyme plants in Farsh Shoeib near St Katherine fell from 1208 to 669, i.e. 44.6 % of the plants disappeared (assumed to have died). Between 2002 and 2010, the condition of plants deteriorated decreasing from 53 % to 25 %; one-third of the surviving plants were <10 % green [28].

Most likely as a result of these local climatic changes, a greater effort had to be made to catch representative numbers of mice for our study - reflected in the significantly higher number of trapping hours in 2008 and 2012, in comparison to 2000 and 2004. Although we are not able to conclude with any degree of certainty if rodent population sizes have actually fallen because of this significant change in their habitats (reduction in water availability and in the acreage of Bedouin gardens), we may nevertheless be certain that at least the population densities of the mouse subpopulations were lower in the latter two surveys. This may have affected the transmission of parasites with consequent lower prevalence - both density-dependent or frequency-dependent, as established convincingly in the long-term studies on cowpox virus transmission in field voles, *Microtus agrestis,* in Kielder forest, UK (reviewed in [3]). Changes in the abiotic factors in the study sites affected not only the host populations but also could have had a direct negative impact on the survival of parasite vectors such as juvenile fleas and ticks, contributing also to overall lower transmission rates.

Generally, the changes observed in the haemoparasite communities of spiny mice in the Sinai were much more pronounced and more diverse than the patterns/trends observed in our other studies on haemoparasites in common and bank voles from central Europe (Poland) [4, 19, 20]. This marked dynamic may reflect a more fragile structure of parasite communities in the hyper-arid environment in Egypt, in comparison to the relatively more predictable abiotic conditions in woodland habitats in central and northern Europe, which show marked seasonal changes but an annual sequence of changes that varies little from year to year.

Although we observed significant temporal changes, nevertheless the site of sampling of the mouse population was always the main factor influencing haemoparasite community structure and many of the differences between the wadis that we observed in 2000 [10] were maintained during the subsequent 12-year-long period of monitoring. Subpopulations from wadis Tlah and Gharaba constituted the main hosts for *T. acomys* and *Hepatozoon* sp., and spread of the latter species to other mouse populations observed in 2008 was only partially successful. It is apparent therefore that ideal conditions for maintaining the host-vector-parasite relationship exist only in these two sites/habitats. As we reported in our earlier paper [10], fleas (*Parapulex chephrensis)*, the most likely vectors of *Trypanosoma* [41] and *Hepatoozon,* were found mostly on mice from Wadis Tlah and Gharaba (unpublished observations for 2012 confirm the published data from 2000, reported in [10]). In 2000 no fleas were found on mice from W. Gebal, and only two mice from W. El Arbaein were found with fleas. The overall prevalence of *T. acomys* among mice with its likely flea vector was 44.8 % compared with just 12 % among mice without fleas and there was a weak but statistically significant overall association between prevalence of *T. acomys* and flea infestation [10]. Similarly, in 2000 the prevalence of *Hepatozoon* sp. among mice with fleas was 41.4 %, in contrast to 16.8 % among mice without fleas. However, when the site, sex and age effects were controlled for, no significant association was evident between these taxa.

Comparing haemoparasite communities between the host subpopulations from the four wadis, spiny mice from W. Tlah consistently showed the highest mean species richness, total species richness and the highest prevalence of all haemoparasites. This site experienced also the least change in habitat structure over the 12-year period and the lowest loss of arable land. Wadi Tlah consists of a deep, narrow valley with steep cliffs on both sides, with ample shaded areas, well-maintained gardens and few apparent signs of aridification because it drains most of the high-mountain region, so any rainfall will percolate through it. The prevalence of *T. acomys* underwent almost no change in mice from this wadi. In contrast, by the end of our study period mice from the high-altitude W. Gebal showed the lowest mean species richness and lowest prevalence of all haemoparasites. This is in agreement with previous studies on haemoparasites from *A. dimidiatus* from this wadi, and is also consistent with studies on intestinal micro- and macroparasites and ectoparasites [9, 10]. Mice from W. Gebal have consistently revealed an impoverished parasite fauna, lacking both the flea *P. chephrensis* and *T. acomys*, in addition to the absence of the dominant nematode *Protospirura muricola* [9]. However, this wadi constituted the main focus for the transmission of a novel *Babesia* species- *B. behnkei*– discovered in Wagner’s gerbil *D. dasyurus* in 2004 and recently described [42]. This high-altitude wadi experiences the most extreme abiotic conditions and greatest degree of aridification/desertification: by 2008 about half of Bedouins’ gardens in this wadi had been abandoned and in 2012 even the remaining trees and bushes were extensively desiccated and damaged by grazing feral donkeys. In contrast, mice from the low-level W. Gharaba, which has greater exposure to direct sunlight and is considerably warmer than the other three wadis, were heavily infected with *P. muricola*, *P. chephrensis* and *T. acomys* [9, 10]. As with W. Gebal, this site has experienced severe shortage of water with resulting increased aridity, and this change in climatic conditions is reflected in reduced prevalence and abundance of both *Haemobartonella* sp. and *T. acomys*, but interestingly not *Hepatozoon*. Wadi El Arbaein is the site that is most affected by human activities, with a large town (St Katherine) localized at its mouth, and it experiences extensive exposure to livestock, mainly goats and camels, but also cats and dogs. This is also the wadi with the highest tourist activity. During the 12-year-long period the town has grown and developed (i.e. construction of new paved roads, lighting on the streets) but has also been affected by drought, the level of ground water having fallen alarmingly over this period (from 7 m to about 25 m in Fox Camp). The construction of new water storage tanks and water pipes for the provision of water for the city from the coast had not been successfully completed by 2012. Perhaps not surprisingly therefore, the mice from W. El Arbaein showed the lowest prevalence of haemoparasites in the early surveys and a variable pattern of haemoparasite species richness and prevalence, generally as in the mice from W. Gebal. Interestingly, as in the mice from W. Gebal, *H. acomys* was introduced to the mice in this wadi in 2004 and then increased in prevalence and persisted through to 2012, creating a third location for the occurrence of this parasite among spiny mice in our study sites.

As we had predicted, host sex has no detectable influence on the haemoparasite community, and this is consistent with many earlier studies [3, 22–24], including our work in Egypt and Poland [4, 10, 20, 21]. However, there were two contrasting patterns in relation to host age. For *Babesia* and *Haemobartonella*, the highest prevalence or abundance were observed in young animals, but for *Trypanosoma* and *Hepatozoon* the highest infection parameters were from adults (class 2 or 3). The former pattern of increasing likelihood of carrying infection with increasing host age is consistent with the idea that the longer the mouse lives, the higher the probability of being infested by an infected vector and hence of contracting the infection. Most of the parasites identified in this study typically cause long subclinical infections in their natural hosts [37, 42], and thus the proportion of animal carrying infection increases with host age, peaking among the older animals. The latter pattern of peak prevalence in young animals, as observed in the case of *T. acomys* and *Hepatozoon* sp., can have two explanations. One possibility is vertical transmission resulting in congenital or neonatal (i.e. transmitted by nest ectoparasites) infection and this combined with the high susceptibility of young naïve individuals to infections, and followed by acquired immunity reducing prevalence among the older sectors of the population. Vertical transmission and congenital infections have been confirmed for *Babesia* spp. [43, 44], *Hepatozoon balfouri* [40] and *Haemobartonella* (*Mycoplasma*) [45].

Rodent trypanosomes are known to generate potent sterilizing immunity. Thus the declining prevalence of *T. acomys* in the oldest age class, was not unexpected given that this species probably generates life-long immunity just like *T. lewisi* and *T. musculi* to which it is closely related. The long patent period explains the high prevalence of infection with *T. acomys* in mice of age class 2, and the decline in the oldest mice (age class 3) suggests either the action of some form of immunity in laterlife or selective mortality of infected older mice. An immune response certainly occurs in *T. lewisi* and *T. musculi*, and is suggested by the data of Abdallah et al. [37] for *T. acomys*, which show clearance from peripheral blood after 150 days, although a mechanism for this has not yet been identified.

## Conclusions

Haemoparasite communities varied markedly between four subpopulations of spiny mice living in isolated wadis and displayed long-term trends, most likely associated with a changing environment driven by decade-long drought. As predicted, external factors (site and year of study) had a much greater influence on parasite communities than intrinsic factors (host age or sex).

## Additional files

Additional file 1: Nucleotide sequences and annealing temperature of the primers used for polymerase chain reaction (PCR). (DOCX 17 kb) Additional file 2: Alignment of two 18S rDNA *Hepatozoon* variants with the two most similar sequences of *Hepatozoon* from the GenBank database. (DOCX 74 kb) Additional file 3: Alignment of two 18S rDNA *Trypanosoma* variants with the most similar sequences of *Trypanosoma* from the GenBank database. (DOCX 104 kb)
