# Supplementary material for: Long-term spatiotemporal stability and dynamic changes in the haemoparasite community of spiny mice (Acomys dimidiatus) in four montane wadis in the St. Katherine Protectorate, Sinai, Egypt
Source: Parasit Vectors. 2016 Apr 8;9:195. doi: 10.1186/s13071-016-1471-z (PMC4826546; doi:10.1186/s13071-016-1471-z)
Supplement: Additional file 1: — Nucleotide sequences and annealing temperature of the primers used for polymerase chain reaction (PCR). (DOCX 17 kb) [file 13071_2016_1471_MOESM1_ESM.docx]

Supplementary file 1. Nucleotide sequences and annealing temperature of the primers used for polymerase chain reaction (PCR)

| Reference | Product  size (bp) | Annealing temperature (⁰C) | Primers sequence 5ʼ 3ʼ | Primers | Gen /region | Parasite species |
| --- | --- | --- | --- | --- | --- | --- |
| Paziewska et al. 2011 | 333 | 52 | GCACGATTYGCATCATCATTTTCC  CGCATTATGGTCGTATTTGTCC | rpoB F  rpoB R | *rpoB* | *Bartonella* |
| Inokuma et al. 2002 | 660 | 60 | ATACATGAGCAAAATCTCAAC  CTTATTATTCCATGCTGCAG | Hep F  Hep R | 18S rRNA | *Hepatozoon* |
| Noyes et al. 1999 | ~1700  ~622 | 58 | GAAACAAGAAACACGGGAG  CTACTGGGCAGCTTGGA  TGGGATAACAAAGGAGCA  CTGAGACTG-TAACCTCAAAGC | TRY816F TRY816R (external)  SSU561F  SSU561R  (internal) | 18S rRNA | *Trypanosoma* |
