# Supplementary figures and images for: Long-term spatiotemporal stability and dynamic changes in the haemoparasite community of spiny mice (Acomys dimidiatus) in four montane wadis in the St. Katherine Protectorate, Sinai, Egypt
Source: Parasit Vectors. 2016 Apr 8;9:195. doi: 10.1186/s13071-016-1471-z (PMC4826546; doi:10.1186/s13071-016-1471-z)

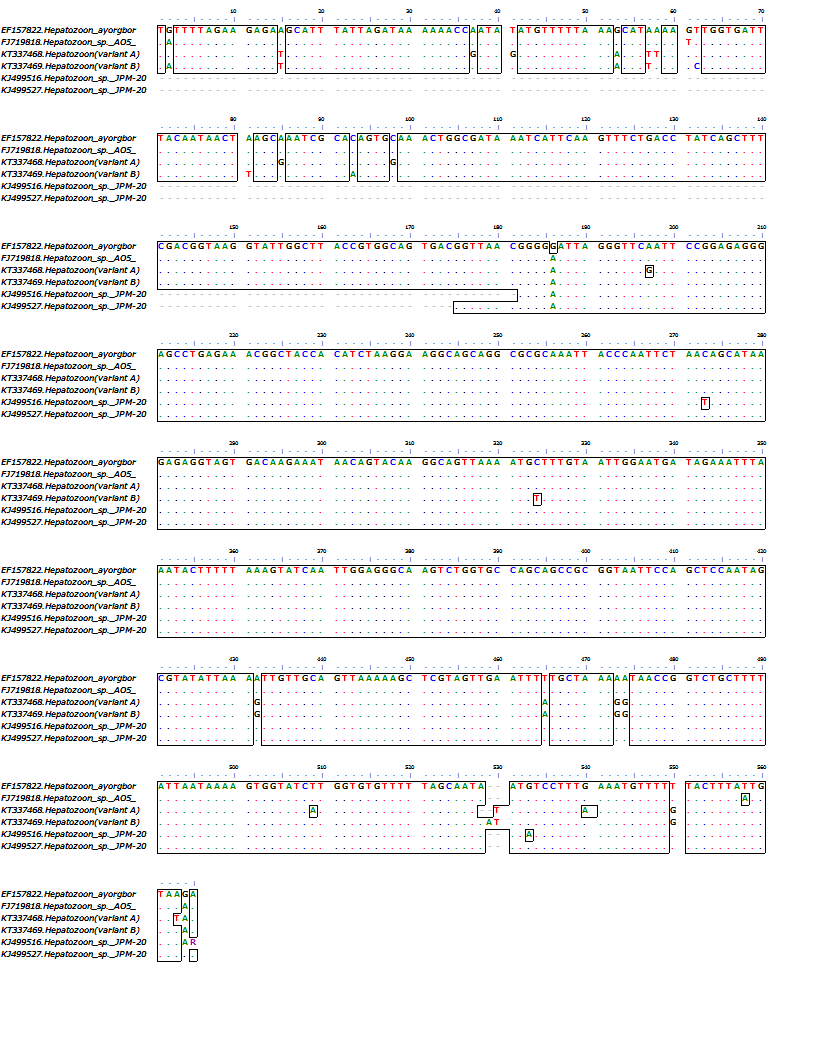

Supplement: Additional file 2: — Alignment of two 18S rDNA Hepatozoon variants with the two most similar sequences of Hepatozoon from the GenBank database. (DOCX 74 kb) [file 13071_2016_1471_MOESM2_ESM.docx]

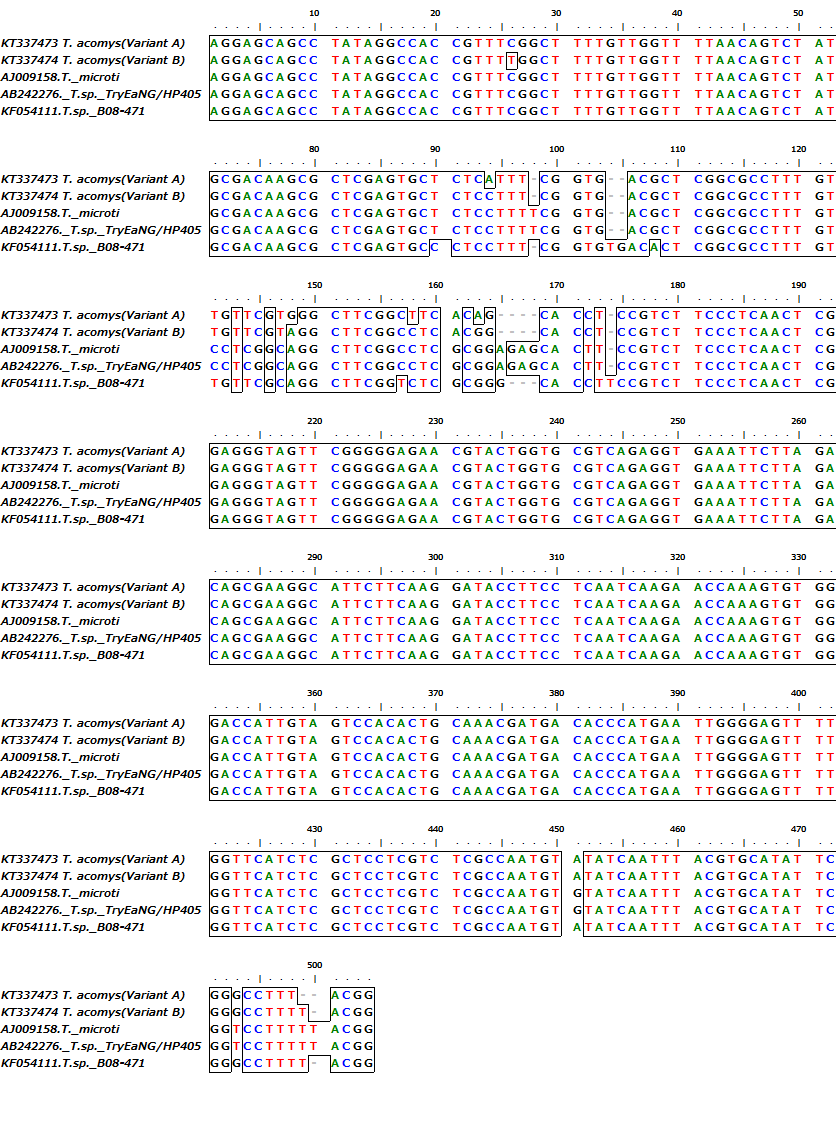

Supplement: Additional file 3: — Alignment of two 18S rDNA Trypanosoma variants with the most similar sequences of Trypanosoma from the GenBank database. (DOCX 104 kb) [file 13071_2016_1471_MOESM3_ESM.docx]
